# Smoking-attributable mortality and years of potential life lost in 16 Brazilian capitals, 2003: a prevalence-based study

**DOI:** 10.1186/1471-2458-9-206

**Published:** 2009-06-26

**Authors:** Paulo CRP Corrêa, Sandhi M Barreto, Valéria MA Passos

**Affiliations:** 1 Hospital Alberto Cavalcanti, Fundação Hospitalar do Estado de Minas Gerais (FHEMIG), Rua Camilo de Brito 636, Bairro Padre Eustáquio, ZIP 30730-540, Belo Horizonte, Brazil; 2 Primeira Companhia Independente, Polícia Militar do Estado de Minas Gerais, Rua Miguel Couto 89, Bairro Retiro, ZIP 30400-000, Nova Lima, Brazil; 3 Department of Preventive and Social Medicine, Faculty of Medicine, Federal University of Minas Gerais (UFMG), Belo Horizonte, Brazil; 4 Department of Internal Medicine, Faculty of Medicine, Federal University of Minas Gerais (UFMG), Belo Horizonte, Brazil

## Abstract

**Background:**

To establish the impact of tobacco smoking on mortality is essential to define and monitor public health interventions in developing countries.

**Methods:**

The Smoking-Attributable Mortality, Morbidity and Economic Costs (SAMMEC) software was used to estimate the smoking attributable mortality (SAM) in 15 Brazilian State Capitals and the Federal District for the year 2003. Smoking prevalence and mortality data of people aged 35 years or older were obtained for each city from the Brazilian Household Survey on Non Communicable Diseases Risk Factors (2002–2003) and from the Brazilian Mortality System (2003), respectively.

**Results:**

In 2003, of the 177,543 deaths of persons aged 35 years and older 24,222 (13.64%) were attributable to cigarette smoking. This total represents 18.08% of all male deaths (n = 16,896) and 8.71% (n = 7,326) of all female deaths in these cities. The four leading causes of smoking-attributable death were chronic airways obstruction (4,419 deaths), ischemic heart disease (4,417 deaths), lung cancer (3,682 deaths), and cerebrovascular disease (3,202 deaths). Cigarette smoking accounted for 419,935 years of potential life lost (YPLL) (279,990 YPLL for men and 139,945 YPLL for women) in the same period.

**Conclusion:**

Tobacco use caused one out of five male deaths and one out of ten female deaths in the sixteen cities in 2003. Four leading causes of smoking attributable deaths (ischemic heart disease, chronic airways obstruction, lung cancer and cerebrovascular disease) accounted for 64.9% of SAM. Effective and comprehensive actions must be taken in order to slow this epidemic in Brazil.

## Background

Scientific evidence of harm caused by smoking has been accumulating for over 200 years, at first in relation to cancers of the lip and mouth, and then in relation to vascular diseases and lung cancer [1]. Epidemiologic studies have estimated disease risks associated with various smoking patterns. At present, nearly 40 diseases or causes of death are known to be positively associated with cigarette smoking [1]. As a result of these findings, in the next 50 years, tobacco use is projected to cause nearly 450 million deaths worldwide [2]. It is expected that by 2020–2030, approximately 7 million deaths attributable to tobacco will occur per year in developing countries [3].

Counting and establishing the causes of deaths is a matter of concern for the public health community. Two of the Millennium Development Goals express targets in terms of mortality, which is also one of the three components of the Human Development Index [4]. Information on deaths is crucial to the planning, implementation and evaluation of public health programs at local, national and international levels.

The number of deaths caused by tobacco use in a population (the smoking-attributable mortality, SAM) can be estimated by different methodologies [5-7]. The population attributable risk (PAR) methodology is the most commonly used [5]. PAR incorporates the prevalence of smoking and the relative risk (RR) associated with various amounts of smoking [5-7]. Adult Smoking-Attributable Mortality, Morbidity and Economic Costs (SAMMEC), an online application developed by the Centers for Disease Control and Prevention (CDC), use attributable risk formulas to estimate the number of deaths from cancer, cardiovascular and respiratory diseases associated with cigarette smoking [8]. SAMMEC has been applied in United States and other countries such as Australia [9], Canada [10] and Spain [11].

In developed countries, data on major health risk factors are regularly obtained from population surveys and morbidity specific registers such as those for cancer. Many developing countries have reasonably reliable data on mortality by cause, but lack population data on the prevalence of risk factors, such as smoking, which are essential to establish public health policy priorities. In Brazil, direct estimates of mortality cannot be made because there is a lack of longitudinal studies on the differential mortality of smokers, former smokers and non-smokers, necessary to provide RR estimates for smoking-related diseases and mortality. Attempts to produce indirect estimates are needed, because an important share of the global tobacco burden falls on developing countries, where 84% of the 1.3 billion current smokers reside [12]. In 2002 smoking-related mortality in Brazil was estimated at around 200,000 a year [13]. In 2003, the Ministry of Health conducted a large population-based survey in 16 Brazilian capitals [14] to assess the prevalence of smoking and several other risk factors. The results of the survey allowed, along with other information, to estimate for the first time the number of smoking attributable deaths and the years of potential life lost (YPLL) in these capitals. In order to assist the reader with interpreting the results we have provided a map of Brazil and the capitals included in the study as an additional file.

## Methods

After considering all the methods that could be used to estimate SAM in Brazil [6], we decided to use the PAR method. A direct estimate of the SAM in Brazil is not possible since the smoking status of decedents is not routinely collected on the death certificate and relative risk estimates for cause specific mortality related to tobacco use in Brazil are not available. The PAR method was selected because it is frequently used throughout the world and because of the availability of a free computational tool on the internet, with relative risks adjusted for age and sex (SAMMEC) [6]. The smoking impact ratio (SIR) method proposed by Peto [15,16] is complex and there is no easy access to technical assistance unlike the SAMMEC method. In addition, the SIR calculation requires lung cancer mortality rates in never smokers, which are not available in Brazil.

SAMMEC was used to calculate age-adjusted SAM rates for persons aged 35 years and older, using age, sex and cause specific mortality rates, current smoking prevalence by age group and sex in the same period [8], and the American Cancer Society's Cancer Prevention Study II (CPS-II) relative risks [17]. The CPS-II is an ongoing prospective study of 1,185,106 residents in United States, aged 30 years or over, for those who, in 1982, had never smoked regularly, and for those who were then current cigarette smokers [15]. The cause-specific RR for a smoking-related disease represents the ratio of mortality among current or former smokers to mortality among those who never smoked [17]. We used these RR estimates because they are robust; the age of initiation of smoking in Brazil and US are similar [18] and both Brazil and U.S experienced a significant decrease in the number of cigarettes smoked per day in relation to what occurred at the beginning of the 90s [19,20].

In the present study, data from the 2000 Demographic Census, according to sex and five-year age group, provided the denominators for mortality rates of persons aged 35 years and over [21]. The 2003 mortality data for 22 adult smoking-related diseases were drawn from the Brazilian Mortality System, available from the Brazilian Health Database (DATASUS) [22]. Mortality rates for each city were then age-standardized using the age distribution of the total Brazilian population in the same year [21]. Deaths were categorized by cause, city of residence, sex and age group. Causes were coded using the International Classification of Diseases (ICD), 10th Revision. Data on deaths from burns or second hand smoke were not available and therefore were not included in the present study.

Smoking prevalence rates for adults aged 35 years or older were obtained from the database of the Brazilian Household Survey on Non-Communicable Diseases Risk Factors and Self-Reported Morbidity [14]. The target population of this survey consisted of individuals aged 15 years or older who lived in 16 Brazilian cities. The survey included all State capitals of the South and Southeast regions; two out of four State capitals of the Central-West region, five out of nine State capitals of the Northeast region and two out of seven State capitals of the North region. Altogether the population of these 16 State capitals accounted for about 20% of the Brazilian total population in 2000 [21]. In this survey, smokers were defined as those who reported, at the time of the interview, smoking cigarettes every day and had smoked at least a hundred cigarettes in their lifetime. In the absence of information on the total number of cigarettes or similar products smoked by non-daily smokers (approximately 10% of total smokers), a consumption equivalent to one cigarette per day was attributed to these non-daily smokers [19]. A total of 23,457 participants were interviewed (10,175 males and 13,282 females) [14]. Current and former smoker rates, by city, sex and two different age groups (35–64 years and ≥ 65 years) were drawn from the survey database using STATA 9.2 software [23].

SAMMEC calculates YPLL multiplying the SAM estimates for each age group, stratified by sex and underlying cause of death by the life expectancy of people at the midpoint of each age range. The resulting numbers for each age group are added to obtain YPLL by sex [8] and group of cause. The total YPLL is the sum of the male and female YPLL for cancer, cardiovascular and respiratory diseases.

Data to estimate YPLL was obtained from the Brazilian Institute of Geography and Statistics (IBGE) [24]. SAMMEC uses life expectancies estimates for five-year age categories from 35–39 to 85 + years. As IBGE does not provide life expectancies for people over 80 years of age [21], we applied the same life expectancy of age group 75–79 to those aged 80 + years.

Our study uses secondary data published and available for use by the Brazilian Ministry of Health. Resolution N° 196/96 on Research Involving Human Subjects states that ethical clearance to perform an analysis based on secondary data in the public domain is not necessary[25].

## Results

The prevalence of current and former smoking by city, sex and two age groups (35–64 years and ≥ 65 years) are shown in Table 1. The prevalence of smokers and ex-smokers are much higher among men, with the latter category being generally higher than the former one, especially among older adults.

**Table 1:** Percentages of current and former smokers by city, gender and two different age groups. Brazil, 2003.

**City**	**Age Category**	**Male**		**Female**	
		**Current Smoker**	**Former Smoker**	**Current Smoker**	**Former Smoker**

Aracaju	35–64	20.95	26.35	15.64	21.33

	65+	21.05	31.58	6.90	0.00

Belém	35–64	28.77	33.33	17.74	18.39

	65+	23.81	42.86	4.55	30.30

Belo Horizonte	35–64	33.49	35.42	20.95	24.41

	65+	13.79	60.34	2.40	20.00

Brasília	35–64	26.42	34.38	19.78	22.86

	65+	12.12	30.30	8.62	18.97

Campo Grande	35–64	21.43	40.48	14.67	21.20

	65+	5.00	45.00	10.00	5.00

Curitiba	35–64	26.00	30.87	21.59	20.13

	65+	13.85	50.77	12.20	17.07

Florianópolis	35–64	28.96	34.43	24.55	16.96

	65+	10.00	56.67	5.77	11.54

Fortaleza	35–64	27.50	35.75	19.66	24.72

	65+	14.75	50.82	4.85	22.33

João Pessoa	35–64	34.74	23.16	15.33	16.33

	65+	12.50	42.50	8.20	13.11

Manaus	35–64	30.08	24.06	16.96	20.76

	65+	22.22	46.67	6.25	29.17

Natal	35–64	26.36	27.13	18.56	18.56

	65+	14.29	50.00	2.38	23.81

Porto Alegre	35–64	32.14	33.93	27.27	25.13

	65+	13.43	64.18	7.14	15.31

Recife	35–64	27.66	32.45	20.00	20.00

	65+	23.68	36.84	5.88	19.12

Rio de Janeiro	35–64	25.68	35.52	22.80	23.30

	65+	16.96	50.89	5.13	11.79

São Paulo	35–64	30.42	33.75	19.87	25.64

	65+	25.00	34.38	9.09	18.18

Vitória	35–64	24.03	32.47	17.20	17.20

	65+	7.69	69.23	8.11	18.92

In 2003, a total of 177,543 deaths of individuals aged 35 years and older (93,431 males; 84,112 females) were reported in the 16 State capitals. From this total, 83,593 deaths were linked to smoking; 27 deaths were assigned to the ICD-10 code F17.2 (tobacco dependence syndrome) but no underlying (or primary) tobacco-related cause was reported. For this reason, these deaths were excluded, thus 83,566 deaths remained for the estimation of SAM. A total of 24,222 deaths (13.64% of all deaths and 28.99% of all smoking-related diseases deaths) were caused by smoking. The male-to-female SAM ratio was equal to 2.31 (Table 2).

**Table 2:** Numbers and percentages of smoking-attributable deaths^1 ^according to disease group and sex among people aged ≥ 35 years in 16 Brazilians cities, 2003.

**Disease Category**	**ICD-10**	**Male (%)**	**Female (%)**	**Total**
**Malignant Neoplasms**				

Lip, Oral Cavity, Pharynx	C00-14	827 (4.89)	97 (1.32)	924 (3.81)

Oesophagus	C15	664 (3.92)	134 (1.83)	798 (3.29)

Stomach	C16	498 (2.95)	86 (1.17)	584 (2.41)

Pancreas	C25	157 (0.93)	137 (1.87)	294 (1.21)

Larynx	C32	533 (3.15)	52 (0.71)	585 (2.42)

Trachea, Lung, Bronchus	C33-34	2632 (15.58)	1050 (14.33)	3682 (15.20)

Cervix Uteri	C53	0 (0)	94 (1.28)	94 (0.39)

Kidney and Renal Pelvis	C64-66, C68	119 (0.70)	0 (0)	119 (0.49)

Urinary Bladder	C67	206 (1.22)	36 (0.49)	242 (1.00)

Acute Myeloid Leukaemia	C92.0	36 (0.21)	10 (0.14)	46 (0.19)

**Sub-total**		5636 (33.36)	1696 (23.15)	7332 (30.27)

				

**Cardiovascular Diseases**				

Ischemic Heart Disease	I20-25	3151 (18.65)	1266 (17.28)	4417 (18.24)

Other Heart Disease	I00-09, I26-28, I30-51	1102 (6.52)	405 (5.53)	1507 (6.22)

Cerebrovascular Disease	I60-69	1863 (11.03)	1339 (18.28)	3202 (13.22)

Atherosclerosis	I70	44 (0.26)	9 (0.12)	53 (0.22)

Aortic Aneurysm	I71	701 (4.15)	297 (4.05)	998 (4.12)

Other Arterial Disease	I72-78	58 (0.34)	39 (0.53)	97 (0.40)

**Sub-total**		6919 (40.95)	3355 (45.80)	10274 (42.42)

				

**Respiratory Diseases**				

Pneumonia, Influenza	J10-18	899 (5.32)	452 (6.17)	1351 (5.58)

Bronchitis, Emphysema	J40-43	603 (3.57)	243 (3.32)	846 (3.49)

Chronic Airway Obstruction	J44	2839 (16.80)	1580 (21.57)	4419 (18.24)

**Sub-total**		4341 (25.69)	2275 (31.05)	6616 (27.31)

				

**Total**		16896 (100)	7326(100)	24222(100)

^1 ^Does not include burn or second hand smoke deaths.

Table 2 presents the number of smoking-attributable deaths by sex grouped into three broad categories: cancer, cardiovascular and respiratory diseases. Cardiovascular diseases were the most frequent cause, responsible for 10,274 of all smoking attributable deaths, followed by cancer (7,332 deaths) and respiratory diseases (6,616 deaths). The four leading specific causes of adult smoking-attributable deaths were chronic airways obstruction (4,419 deaths), ischemic heart disease (4,417 deaths), lung cancer (3,682 deaths) and cerebrovascular disease (3,202 deaths). Combined, these four conditions were responsible for 64.9% of all SAM (15,720/24,222). Males and females differed slightly in the ranking of the four leading causes of smoking attributable deaths. Among males they were: ischemic heart disease (IHD), chronic airways obstruction, lung cancer and cerebrovascular disease. Among females they were chronic airways obstruction, cerebrovascular disease, ischemic heart disease and lung cancer.

Of the total smoking-attributable deaths, 81.1% occurred in six cities: São Paulo (9,201); Rio de Janeiro (5,076); Porto Alegre (1,573); Belo Horizonte (1,394); Curitiba (1,360) and Recife (1,029). Table 3 presents age-adjusted SAM rates per 100,000 Brazilians for these cities. Curitiba had the highest age-adjusted SAM rates, followed by Porto Alegre, São Paulo, Recife, Rio de Janeiro, and Belo Horizonte. Additional files are available for each one of the 16 Brazilian capitals. Each additional file contains 3 Microsoft Excel spreadsheets: spreadsheet 1 shows Mortality Smoking-Attributable Fractions by Sex and Age; spreadsheet 2 displays Smoking-Attributable Mortality, whereas spreadsheet 3 shows Age-Adjusted SAM Rate per 100,000 [additional files 1, 2, 3, 4, 5, 6, 7, 8, 9, 10, 11, 12, 13, 14, 15, 16 and 17].

**Table 3:** Age-adjusted smoking attributable mortality (SAM)^1,2 ^rates per 100,000 inhabitants, according to cause of death in selected cities, Brazil, 2003.

	São Paulo	Rio de Janeiro	Porto Alegre	Belo Horizonte	Curitiba	*Recife*
**Disease Category**						

**Malignant Neoplasms**						

Lip, Oral Cavity, Pharynx	8.2	7.9	6.9	5.8	10.3	*6.9*

Oesophagus	7.7	4.9	10.2	7.4	10.7	*4.1*

Stomach	5.7	3.6	4.4	4.6	4.2	*3.5*

Pancreas	3.0	2.2	2.7	2.2	3.1	*3.2*

Larynx	5.7	4.3	4.8	3.5	4.2	*4.9*

Trachea, Lung, Bronchus	30.1	29.5	48.6	23.4	34.6	*26.2*

Cervix Uteri	0.8	0.8	0.9	0.3	1.0	*1.1*

Kidney and Renal Pelvis	1.2	1.0	2.6	0.4	1.3	*0.9*

Urinary Bladder	2.6	1.8	3.9	1.8	2.5	*1.1*

Acute Myeloid Leukaemia	0.6	0.5	0.3	0.4	0.0	*0.0*

**Sub-total**	**65.6**	**56.5**	**85.3**	**49.8**	**71.9**	***51.9***

						

**Cardiovascular Diseases**						

Ischemic Heart Disease	48.3	31.4	38.3	24.7	45.2	*46.3*

Other Heart Disease	13.6	10.8	11.3	17.1	12.7	*11.3*

Cerebrovascular Disease	27.1	26.5	27.1	22.4	26.6	*30.9*

Atherosclerosis	0.8	0.4	0.0	0.1	0.8	*0.5*

Aortic Aneurysm	11.0	5.0	10.6	7.4	11.9	*8.8*

Other Arterial Disease	1.6	0.7	0.5	0.4	0.9	*0.5*

**Sub-total**	**102.4**	**74.8**	**87.8**	**72.1**	**98.1**	***98.3***

						

**Respiratory Diseases**						

Pneumonia, Influenza	17.2	9.4	7.6	7.9	11.7	*7.5*

Bronchitis, Emphysema	8.1	4.3	11.3	4.0	15.9	*7.8*

Chronic Airway Obstruction	41.2	30.5	54.4	33.1	52.5	*26.8*

**Sub-total**	**66.5**	**44.2**	**73.3**	**45.0**	**80.1**	***42.1***

						

**Total**	**234.5**	**175.5**	**246.4**	**166.9**	**250.1**	***192.3***

^1 ^Among adults aged 35 years and older

^2 ^Does not include burn or second hand smoke deaths.

In the same period, smoking accounted for 279,990 YPLL among men and 139,945 YPLL among women. Cardiovascular diseases were the leading cause of smoking-attributable YPLL, causing 198,167 YPLL (Table 4).

**Table 4:** Smoking-Attributable YPLL^1,2 ^by city, cause of death and sex in 16 Brazilian cities, 2003.

**City**	**Male**	**Female**	**Total**
Aracaju	**2,237**	**925**	**3,162**

Belém	**8,044**	**4,175**	**12,219**

Belo Horizonte	**16,888**	**7,833**	**24,721**

Brasília	**10,385**	**5,831**	**16,216**

Campo Grande	**5,139**	**2,617**	**7,756**

Curitiba	**14,483**	**8,435**	**22,918**

Florianópolis	**3,114**	**1,113**	**4,227**

Fortaleza	**9,196**	**5,681**	**14,877**

João Pessoa	**3,559**	**1,842**	**5,401**

Manaus	**6,142**	**3,051**	**9,193**

Natal	**3,687**	**2,061**	**5,748**

Porto Alegre	**17,677**	**8,422**	**26,099**

Recife	**11,485**	**7,094**	**18,579**

Rio de Janeiro	**58,318**	**29,034**	**87,352**

São Paulo	**107,434**	**50,978**	**158,412**

Vitória	**2,202**	**853**	**3,055**

**TOTAL**	**279,990**	**139,945**	**419,935**

1 Among adults aged 35 years and older.

2 Does not include burn or second hand smoke deaths.

## Discussion

To our knowledge, this is the first study to estimate SAM in Brazil using population based data on smoking prevalence. Cigarette smoking was responsible for 13.6% of all adult deaths in the studied population.

Smoking prevalence in Latin America varies widely, not only between countries but also within countries [26]. The percentage of smokers in Brazil is closer to that in the USA (20.8% in 2004) and Canada (20% in 2005) and much lower than in some Latin American countries such as Cuba (37.2% in 1995) and Argentina (40.4% in 2000) [19]. Although smoking prevalence in Mexico is similar to that of many high-income countries, especially for men (36% for men and 13% for women), nearly half of smokers do not smoke daily, and most of the remaining smoke fewer than five cigarettes per day [27]. The picture is quite different from the one seen in Brazil, where nearly 30% of smokers smoke 20 or more cigarettes per day and approximately 10% of total smokers are non-daily smokers [19].

Differences in SAM largely reflect the stage of the smoking epidemic in each country [26], but the above data show that the number of cigarettes smoked per day also plays a role. A larger proportion of deaths in people aged ≥ 35 years has been found in the nearby country of Argentina (16% vs. 13.6%) with a male-to-female SAM ratio equal to 2.50 (21% of death among males and 10% among females) [28], slightly higher than the ratio found in Brazil (2.31). In South Africa, also a developing country, smoking accounted for 8.0 to 9.0% of deaths, with three times as many deaths occurring in males compared with females [29].

The SAM in Mexico in 2004 is much lower than the one observed in this work (5.2% of total deaths; 6.0% in men and 4.3% in women) [27]. The largest proportional effect of tobacco smoking on mortality and burden of disease was in the wealthiest regions of Mexico, particularly the Northern region, where it was responsible for 9% of deaths [27]. Because prevalence of smoking in Mexico is higher than in Brazil, such difference in SAM is likely to reflect, at least in part, to the lesser amount of cigarettes smoked daily in Mexico [27].

As seen in Brazil, males and females differed in the ranking of the leading causes of smoking attributable deaths, but the causes were different from ours. While in Argentina the two top killers among men were lung cancer (22%) and cerebrovascular disease (18%) [28], in Brazil they were ischemic heart disease (18.7%) and respiratory diseases (16.8%), followed by lung cancer (15.6%). Among Argentinean females, the leading cause is other heart disease (21%) [28], while in Brazil chronic airway obstruction alone is responsible for 21%.

Combining the four leading causes of smoking attributable deaths in the 16 cities in 2003 – ischemic heart disease, chronic airways obstruction, lung cancer, and cerebrovascular disease – account for 64.9% of the SAM. These diseases are among the most important causes of death in the country. In 2003, for instance, cardiovascular diseases, cancer and respiratory diseases together were responsible for 58% of all adult deaths in Brazil [30]. Thus, these results suggest that a large proportion of these deaths would be prevented by further reductions in smoking prevalence.

Our findings suggest that cigarette smoking accounted for 419,935 YPLL for adults in the 16 cities in 2003. Cardiovascular diseases were the leading cause of smoking-attributable YPLL. YPLL emphasizes the causes of death occurring in younger persons, thus assisting health policy makers in identifying health priorities.

Important issues, such as the quality and coverage of the cause-of-death statistics, need to be considered when using mortality data to evaluate the health situation of a country [31]. Mathers et al analyzed the death registration system of 115 countries to determine the percentage of causes of deaths coded as unknown and ill-defined [32]. Based on these results, data quality for Brazil was categorized as medium. However, the Brazilian Mortality System is far better in the State capitals and in the South and Southeast regions of the country and has improved considerably in recent years. Thus, the estimates of SAM in the cities located in these two regions are likely to express the actual number of smoking attributable deaths. On the other hand, in the seven cities of the North and Northeast, the estimated number of smoking attributable deaths is probably lower than the real one, due to the greater percentage of deaths classified as unknown or ill defined.

The list of smoking-attributable diseases in SAMMEC does not include colorectal cancer among the malignancies. Studies in various populations show an association between tobacco use and colorectal cancer [33,34]. Recent Nurse's Health Study evidence found that current smoking was associated with an increased risk of colorectal cancer mortality [35]. The addition of colorectal cancer to the list of tobacco-associated malignancies would further increase the SAM and YPLL estimates presented in our work. The SAM and YPLL described in this report also represent a conservative estimate because the calculations did not include deaths from cancers at unspecified sites. Two recent meta-analyses discuss the potential association between smoking and mortality from tuberculosis (TB) [36,37], but this contribution is still controversial, so TB has not been included in the present study.

Another question that needs consideration when interpreting SAM is the different induction periods between tobacco exposure and disease manifestation and death, which can reach two to three decades for some types of cancer. The use of the CDC method in calculating SAM requires the assumption of a steady state of smoking prevalence and mortality risks [38]. In periods of fast decline in smoking prevalence, the attributable-fraction methodology will tend to understate the number of deaths caused by smoking [39], because the deaths occurred in one year results from exposure occurring in the past, when prevalence was much higher. Between 1989 and 2003, the estimated prevalence of smokers among the Brazilian adult population declined approximately 35%, going from 34.8% in 1989 to 22.4% in 2003 or an average reduction of 2.5% per year [19]. As a consequence of such decline, the prevalences of smoking applied in the present study are lower than the ones at the relevant induction periods for most causes included by SAMMEC. Thus, the SAM estimates are probably conservative and the impact of this smoking decline in mortality will be felt only after twenty or more years from now.

Translating attributable risk into a number of excess deaths that would not have occurred had the exposure not existed relies on the assumptions that CPS-II relative risks used in the calculation of SAM are applicable to the Brazilian population and that confounding by other risk factors does not introduce a significant bias. However, CPS-II risks are based on large samples of volunteers and friends of the American Cancer Society, a predominantly affluent portion of the US population, including physicians and health care workers and their spouses. For instance, a study found that CPS-II samples differ in many aspects from the US general population [40]. In respect to Brazil, such differences are expected to be magnified, both in relation to smoking patterns and to the distribution of other relevant confounding factors (such as alcohol in relation to some cancers) for the diseases included in SAM. Furthermore, educational and socio-economic levels are important variables associated with cigarette smoking and mortality and are certainly not the same in CPS-II and Brazilian populations. However, it is difficult to value the effect of such differences in our estimates. Studies carried out in the USA that estimated the effect of confounding from these variables in the calculation of the mortality attributable to smoking resulted in an overestimation of only 1%–2.5% [41]. In Spain, the use of CPS-II risks seems to have underestimated SAM [11]. In Brazil, we do not have any study which has estimated such effect, but we can further conjecture about the bias introduced by the use of CPS-II RR. Peto *et al *observed that the level of lung cancer mortality compared with never-smokers is an indicator of the accumulated hazard of smoking and the "maturity" of the smoking epidemic in a population [15]. Two important facts concerning the tobacco epidemic in Brazil should be considered: 1) the country experienced a large increase in consumption of cigarettes one decade later than the United States and the United Kingdom [42] and 2) the level of consumption per capita was always much lower in Brazil than in the US, Canada and European Union countries such as France, Germany and Italy, even at its pick in the 1980s [42]. It follows that RR in Brazil is expected to be smaller than CPS II relative risks because of the maturation factor; thus the use of CPS II relative risks has overestimated mortality in this study. Environmental factors interacting with smoking are different in Brazil, compared to the U.S.: levels of outdoor and indoor pollution, passive smoking, silica and asbestos use could modify the effects of smoking in Brazilian population in a non-multiplicative way [15]. Nonetheless, CPS-II relative risks are based on very large samples and have been applied to other populations; therefore were the best estimates we could use.

The 16 cities studied had altogether 32,851,808 inhabitants in 2000, 19.34% of Brazil's total population (169, 872, 856 inhabitants) [21]. Despite considering that over 80% of the Brazilian population is urban, and the vast majority live in large population centres [21] we cannot extrapolate with certainty that the results of the present study apply to the whole country. Brazil is a continental country in which smoking prevalence varies both between and within Brazilian states. For instance, in 2003 the smoking prevalence in Porto Alegre – capital of Rio Grande do Sul state – was 25.2% (CI 95% 22.4–28.1) [14,43]. The prevalence by sex and two age groups were shown in Table 1. A survey on risk factors for coronary artery disease conducted in 19 cities in Rio Grande do Sul in 1999–2000 showed that the smoking prevalence was 33.9% (CI 95% 31.0–36.8) [44]. Not only smoking prevalence was lower in the capital as compared to the other cities, but also smoking intensity was lower: 13% of the smokers in Porto Alegre consumed more than twenty cigarettes per day [14], contrasting with the 52.5% in the other cities [44].

Lopez et al proposed the tobacco pandemic model in 1994 [45]. This is a four-stage model defined by changes in three variables: prevalence of adult smoking, consumption of tobacco (the amount smoked per adult in a given period) and Smoking Attributable Mortality. This model shows that the lag time between the rise in smoking prevalence and the rise in SAM is about three-to-four decades [45]. Different tobacco control activities would be necessary to tackle each of the four stages of the cigarette epidemic [45]. As a result of the Brazilian National Tobacco Control Program (NTCP), social acceptance of smoking is declining in the country overall. Brazil has a comprehensive tobacco control policy, which includes bans on tobacco advertising, promotion and sponsorship; health warnings on packages; ban on over-the-counter sales to under 18 year-olds, prohibition of smoking in public establishments and health promotion and education programs in schools. Another important way to address the problem of tobacco use is to provide access to effective treatments. Percentages of former smokers are the best measure of smoking cessation at a population level [46]. The prevalence of smoking in Brazil has declined substantially between 1989 and 2003 [19,43] and a high smoking cessation index (number of ex-smokers divided by the number of smokers plus the number of ex-smokers) in the 16 cities varied from 44% in João Pessoa (northeastern Brazil) to 58% in Campo Grande (west-central Brazil) [14,43]. These figures are very similar to those found in the United States in 2002 [47]. Success in reducing smoking prevalence is likely to lower the burden of SAM in future decades [29]. However, it is important to highlight that the decline in smoking prevalence was less marked among lower socioeconomic strata and the reduction in the mean number of cigarettes smoked per day (from 13.3 to 11.6) was modest [19]. This must be addressed in order to prevent an increase in health related inequities in the country.

Another important indicator of improvement in tobacco control is the decline in proportion of household expenditure on tobacco: from 3% in 1995–1996 to 2% in 2002–2003 [42]. The annual consumption of cigarettes per capita among people older than 15 years also decreased from 1,950 cigarettes per capita in 1986 to 1,337 in 1998, and further to 1,194 in 2001 [48]. This data allows the 16 capitals of the country to be categorized at stage III of the tobacco pandemic model [45].

A number of considerations regarding the Brazilian NTCP must be addressed. Firstly, unlike the U.S., Canada and other countries, the smoke-free indoor movement is only incipient in Brazil. The number of public and work places which restrict or do not allow smoking is still very limited. Recent data from the Global Youth Tobacco Survey (GYTS) in 16 Brazilian State capitals and the Federal District showed that exposure to second hand smoke (SHS) in public places varied from 41.9% in Salvador to 62.2% in Porto Alegre [49]. Another recent study showed that Brazil ranked third in the rates of tobacco use during pregnancy, with Uruguay leading (18.3%), followed by Argentina (10.3%) and Brazil (6.1%) [50]. Female-driven educational campaigns and bans on indoor smoking are important cost-effective tobacco control policies [51,52] that need to be pursued in Brazil.

## Conclusion

This is the first study estimating the smoking attributable mortality for Brazil. The classic tobacco pandemic model explains why, despite its decreasing prevalence, smoking still causes one out of five male deaths and one out of ten female deaths from all causes in 16 Brazilian capitals in 2003. The present mortality burden attributable to smoking in the 16 Capitals studied is approximately 25,000 deaths or more, and almost half a million years of potential life (279,990 years in men and 139,945 in women) were lost as a result. This reflects only part of the impact of smoking on the health of the Brazilian population, since morbidity, disability and costs associated with this behavior have not been analyzed.

In 2002, Brazil was categorized as a country at stage II in the classic model of the tobacco pandemic [53]. These results, taken together with the declining annual Brazilian consumption of cigarettes per capita and the degree of social denormalization of cigarette smoking in Brazil, allows us to classify the sixteen studied capitals at stage III in the tobacco pandemic model.

There is a lack of information on smoking-attributable mortality from developing countries. The current estimates of SAM and YPLL in Brazilian society are unacceptable. Despite significant progress in tobacco control in Brazil in recent years, further policies and interventions aimed at reducing SAM need to be implemented. These include enforcing existing laws that prohibit single cigarette sales, more effective use of price instruments, the creation and enforcement of 100% smoke-free environments, further restrictions on point-of-sale tobacco advertising and strategies to encourage smoking cessation.

## Competing interests

The authors declare that they have no competing interests.

## Authors' contributions

The present work is part of the MSc dissertation of PCRPC at the Postgraduate Program in Public Health of the Federal University of Minas Gerais, supervised by SMB and VMAP. The three authors conceived the study and contributed to data analysis and the writing of this paper. All authors read and approved the final manuscript.

## Pre-publication history

The pre-publication history for this paper can be accessed here:



## Supplementary Material

Additional file 1 **Map of Brazil, its regions and the location of studied capitals**. file containing 2 slides: the first slide is the map of Brazil with its five regions; the second shows the location of the capitals included in the study. Click here for file

Additional file 2 **Aracaju**. Data for the city of Aracaju in 2003, this file contains three spreadsheets: Mortality Smoking-Attributable Fractions (SAF) by Sex and Age, Smoking-attributable mortality (SAM) and Age-Adjusted SAM Rate per 100,000. Click here for file

Additional file 3 **Belém**. Data for the city of Belém in 2003, this file contains three spreadsheets: Mortality SAF by Sex and Age, SAM and Age-Adjusted SAM Rate per 100,000. Click here for file

Additional file 4 **Belo Horizonte**. Data for the city of Belo Horizonte in 2003, this file contains three spreadsheets: Mortality SAF by Sex and Age, SAM and Age-Adjusted SAM Rate per 100,000. Click here for file

Additional file 5 **Brasília**. Data for the city of Brasilia in 2003, this file contains three spreadsheets: Mortality SAF by Sex and Age, SAM and Age-Adjusted SAM Rate per 100,000. Click here for file

Additional file 6 **Campo Grande**. Data for the city of Campo Grande in 2003, this file contains three spreadsheets: Mortality SAF by Sex and Age, SAM and Age-Adjusted SAM Rate per 100,000. Click here for file

Additional file 7 **Curitiba**. Data for the city of Curitiba in 2003, this file contains three spreadsheets: Mortality SAF by Sex and Age, SAM and Age-Adjusted SAM Rate per 100,000. Click here for file

Additional file 8 **Florianópolis**. Data for the city of Florianópolis in 2003, this file contains three spreadsheets: Mortality SAF by Sex and Age, SAM and Age-Adjusted SAM Rate per 100,000. Click here for file

Additional file 9 **Fortaleza**. Data for the city of Fortaleza in 2003, this file contains three spreadsheets: Mortality SAF by Sex and Age, SAM and Age-Adjusted SAM Rate per 100,000. Click here for file

Additional file 10 **João Pessoa**. Data for the city of João Pessoa in 2003, this file contains three spreadsheets: Mortality SAF by Sex and Age, SAM and Age-Adjusted SAM Rate per 100,000. Click here for file

Additional file 11 **Manaus**. Data for the city of Manaus in 2003, this file contains three spreadsheets: Mortality SAF by Sex and Age, SAM and Age-Adjusted SAM Rate per 100,000. Click here for file

Additional file 12 **Natal**. Data for the city of Natal in 2003, this file contains three spreadsheets: Mortality SAF by Sex and Age, SAM and Age-Adjusted SAM Rate per 100,000. Click here for file

Additional file 13 **Porto Alegre**. Data for the city of Porto Alegre in 2003, this file contains three spreadsheets: Mortality SAF by Sex and Age, SAM and Age-Adjusted SAM Rate per 100,000. Click here for file

Additional file 14 **Recife**. Data for the city of Recife in 2003, this file contains three spreadsheets: Mortality SAF by Sex and Age, SAM and Age-Adjusted SAM Rate per 100,000. Click here for file

Additional file 15 **Rio de Janeiro**. Data for the city of Rio de Janeiro in 2003, this file contains three spreadsheets: Mortality SAF by Sex and Age, SAM and Age-Adjusted SAM Rate per 100,000. Click here for file

Additional file 16 **São Paulo**. Data for the city of São Paulo in 2003, this file contains three spreadsheets: Mortality SAF by Sex and Age, SAM and Age-Adjusted SAM Rate per 100,000. Click here for file

Additional file 17 **Vitória**. Data for the city of Vitória in 2003, this file contains three spreadsheets: Mortality SAF by Sex and Age, SAM and Age-Adjusted SAM Rate per 100,000. Click here for file
