# Supplementary material for: Smoking-attributable mortality and years of potential life lost in 16 Brazilian capitals, 2003: a prevalence-based study
Source: BMC Public Health. 2009 Jun 26;9:206. doi: 10.1186/1471-2458-9-206 (PMC2711948; doi:10.1186/1471-2458-9-206)
Supplement: Additional file 1 — Map of Brazil, its regions and the location of studied capitals. file containing 2 slides: the first slide is the map of Brazil with its five regions; the second shows the location of the capitals included in the study. [file 1471-2458-9-206-S1.ppt]

## Slide 1
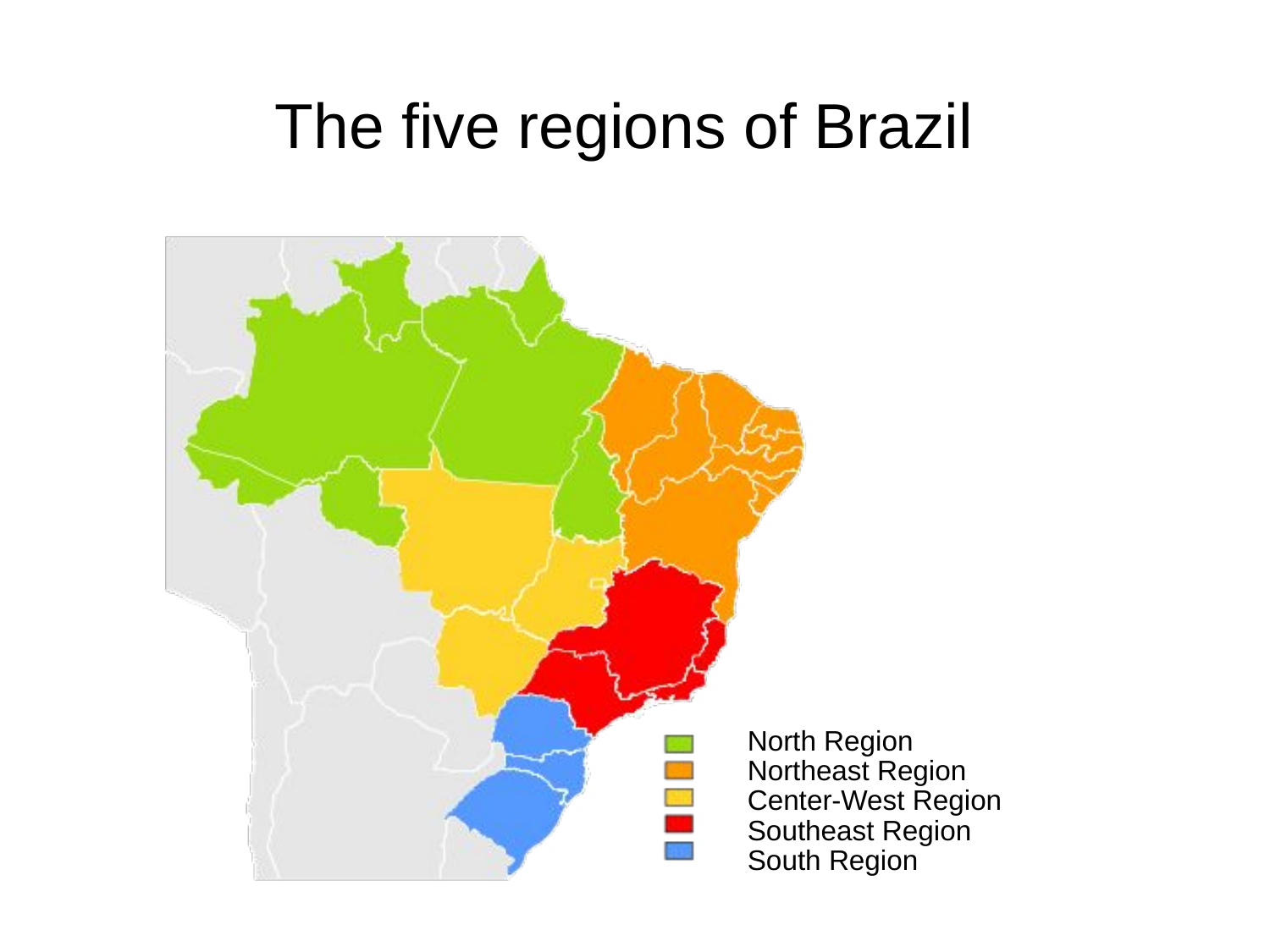

# The five regions of Brazil
North Region
Northeast Region
Center-West Region
Southeast Region
South Region

## Slide 2
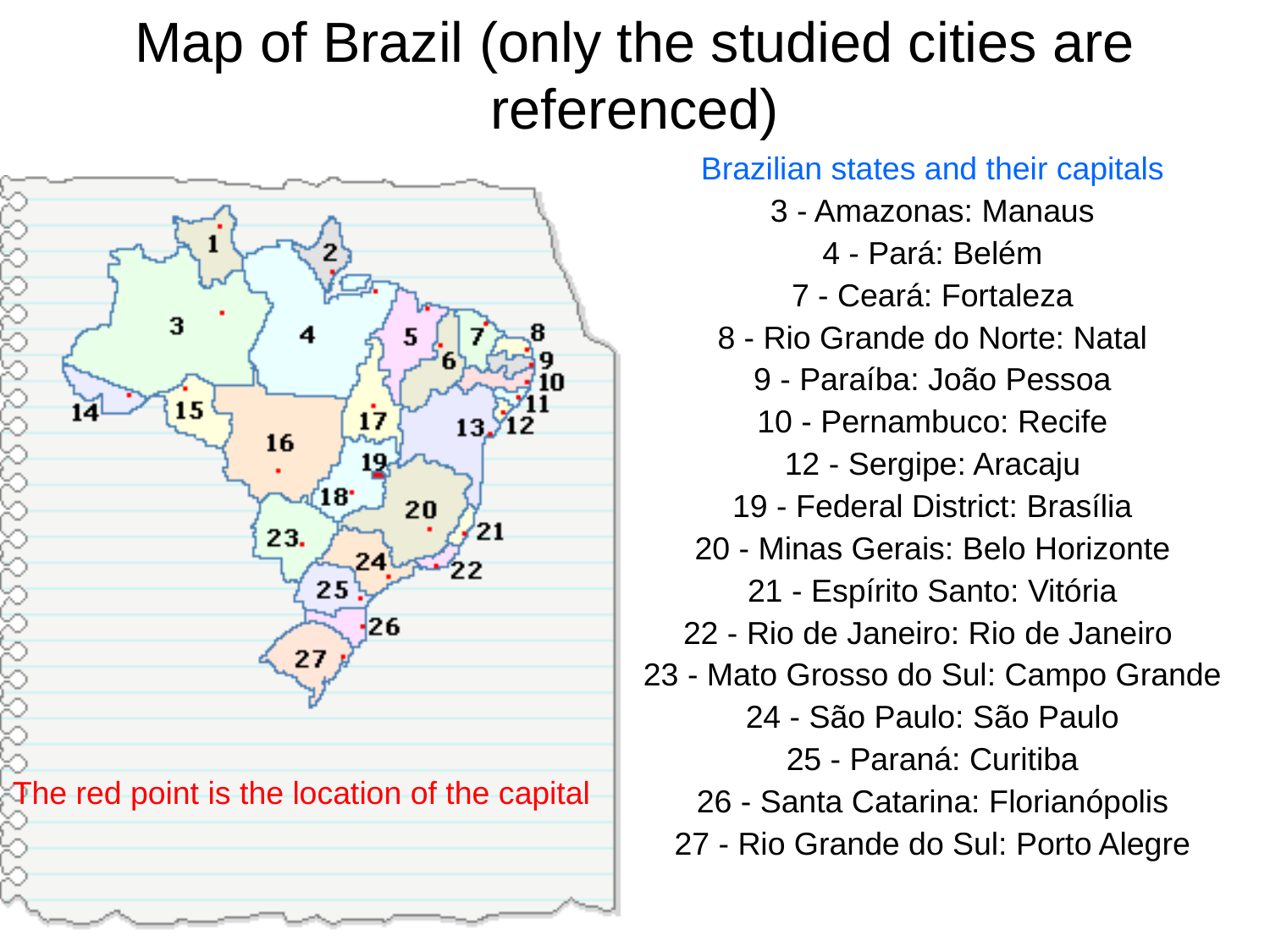

# Map of Brazil (only the studied cities are referenced)
Brazilian states and their capitals
3 - Amazonas: Manaus
4 - Pará: Belém
7 - Ceará: Fortaleza
8 - Rio Grande do Norte: Natal
9 - Paraíba: João Pessoa
10 - Pernambuco: Recife
12 - Sergipe: Aracaju
19 - Federal District: Brasília
20 - Minas Gerais: Belo Horizonte
21 - Espírito Santo: Vitória
22 - Rio de Janeiro: Rio de Janeiro
23 - Mato Grosso do Sul: Campo Grande
24 - São Paulo: São Paulo
25 - Paraná: Curitiba
26 - Santa Catarina: Florianópolis
27 - Rio Grande do Sul: Porto Alegre
The red point is the location of the capital
